# Fratricide activity of MafB protein of *N. meningitidis* strain B16B6

**DOI:** 10.1186/s12866-015-0493-6

**Published:** 2015-08-05

**Authors:** Jesús Arenas, Vincent de Maat, Laura Catón, Massis Krekorian, Juan Cruz Herrero, Flavio Ferrara, Jan Tommassen

**Affiliations:** Department of Molecular Microbiology, Utrecht University, Padualaan 8, 3584 CH Utrecht, The Netherlands

**Keywords:** Adhesin, Anti-toxin, Contact-dependent growth inhibition, MafB, *Neisseria meningitidis*, Toxin, Two-partner secretion

## Abstract

**Background:**

*Neisseria meningitidis* is an inhabitant of the mucosal surfaces of the human nasopharynx. We recently demonstrated that the secreted meningococcal Two-partner secretion protein A (TpsA) is involved in interbacterial competition. The C-terminal end of the large TpsA protein contains a small toxic domain that inhibits the growth of target bacteria. The producing cells are protected from this toxic activity by a small immunity protein that is encoded by the gene immediately downstream of the *tpsA* gene. Further downstream on the chromosome, a repertoire of toxic modules, designated *tpsC* cassettes, is encoded that could replace the toxic module of TpsA by recombination. Each *tpsC* cassette is associated with a gene encoding a cognate immunity protein.

**Results:**

Blast searchers using the toxic domains of TpsA and TpsC proteins as queries identified homologies with the C-terminal part of neisserial MafB proteins, which, for the rest, showed no sequence similarity to TpsA proteins. On the chromosome, *mafB* genes are part of genomic islands, which include cassettes for additional toxic modules as well as genes putatively encoding immunity proteins. We demonstrate that a MafB protein of strain B16B6 inhibits the growth of a strain that does not produce the corresponding immunity protein. Assays in *E. coli* confirmed that the C-terminal region of MafB is responsible for toxicity, which is inhibited by the cognate immunity protein. Pull-down assays revealed direct interaction between MafB toxic domains and the cognate immunity proteins.

**Conclusions:**

The meningococcal MafB proteins are novel toxic proteins involved in interbacterial competition.

**Electronic supplementary material:**

The online version of this article (doi:10.1186/s12866-015-0493-6) contains supplementary material, which is available to authorized users.

## Background

The cell envelope of Gram-negative bacteria consists of two membranes, the inner and outer membrane, which are separated by the periplasm containing a peptidoglycan layer. Different transport machineries have evolved for delivering proteins from their site of synthesis in the cytoplasm to the bacterial surface and beyond. One of them is the two-partner secretion (TPS) system, which is broadly used in many Gram negatives for the specific secretion of usually very large proteins, generically called TpsA [1]. After transport across the inner membrane via the Sec system, the TpsA protein is translocated across the outer membrane via a dedicated transporter, called TpsB. Recognition between the proteins is mediated by a specific N-terminal domain of ~300 amino acid residues in TpsA, the TPS domain, which interacts with a periplasmically exposed domain of the outer-membrane-embedded TpsB protein [1].

In several microorganisms, including *Escherichia coli* and *Neisseria meningitidis*, TpsA functions in inhibiting the growth of related bacteria in competition for the same niche in a process called contact-dependent growth inhibition (CDI) [2–4]. In the proposed model [2], the surface-exposed TpsA interacts with a conserved receptor, BamA, on a target cell, after which a small C-terminal part of TpsA is proteolytically released and transported into the target cell in a process that also requires the inner-membrane protein AcrB [5]. An alternative import pathway requires the interaction of the toxin with the F pilus and pilus retraction [6]. In the target cell, the toxin, can exert different toxic activities, *e.g.* as a DNase or an RNase. The gene immediately downstream of *tpsA*, called *tpsI*, encodes a small immunity protein that protects the producing cell against the toxic activity of the C-terminal domain of TpsA. On the chromosome, the *tpsB*, *tpsA* and *tpsI* genes are present on genetic islands often additionally containing a number of *tpsC* cassettes [4]. These *tpsC* cassettes potentially encode N-terminally truncated TpsA proteins, which, however, present an entirely different toxic module at the C terminus. Each *tpsC* cassette is associated with a cognate *tpsI* gene. Because of the N-terminal truncation, these putative TpsC proteins lack the sequences necessary for secretion and it is not sure whether they are expressed. However, the *tpsC* cassettes can recombine with the *tpsA* locus, thereby replacing the toxic module present at the C terminus of TpsA [4]. Thus, TpsA constitutes an interbacterial competition system that can use a broad repertoire of toxic modules.

Other secretion systems found in Gram-negative bacteria are also meant for inhibiting competing bacteria or even eukaryotic cells. Examples include RhsA (rearrangement hot spot) of *E. coli* [7] or the broadly distributed Type VI secretion system [8]. These growth inhibition systems present similar toxic modules at the C terminus of the exported proteins as found in the TpsA proteins, but show no further sequence similarity with TpsA, consistent with a different secretion mechanism. In the present study, we demonstrate that the MafB proteins of *Neisseria* spp., previously thought to function as adhesins [9], present similar toxic modules at their C terminus as the TpsA proteins, but show no further sequence similarity to TpsA. We demonstrate that these MafB proteins represent a novel growth inhibition system in the meningococcal strain B16B6 that functions in interbacterial competition. Whilst this manuscript was in preparation, another study of the MafB proteins of *Neisseria* spp. was published [10]. For clarity, we have adopted the nomenclature for the Maf proteins of that study.

## Results

### Structural organization of meningococcal Maf islands

**Fig. 1:**
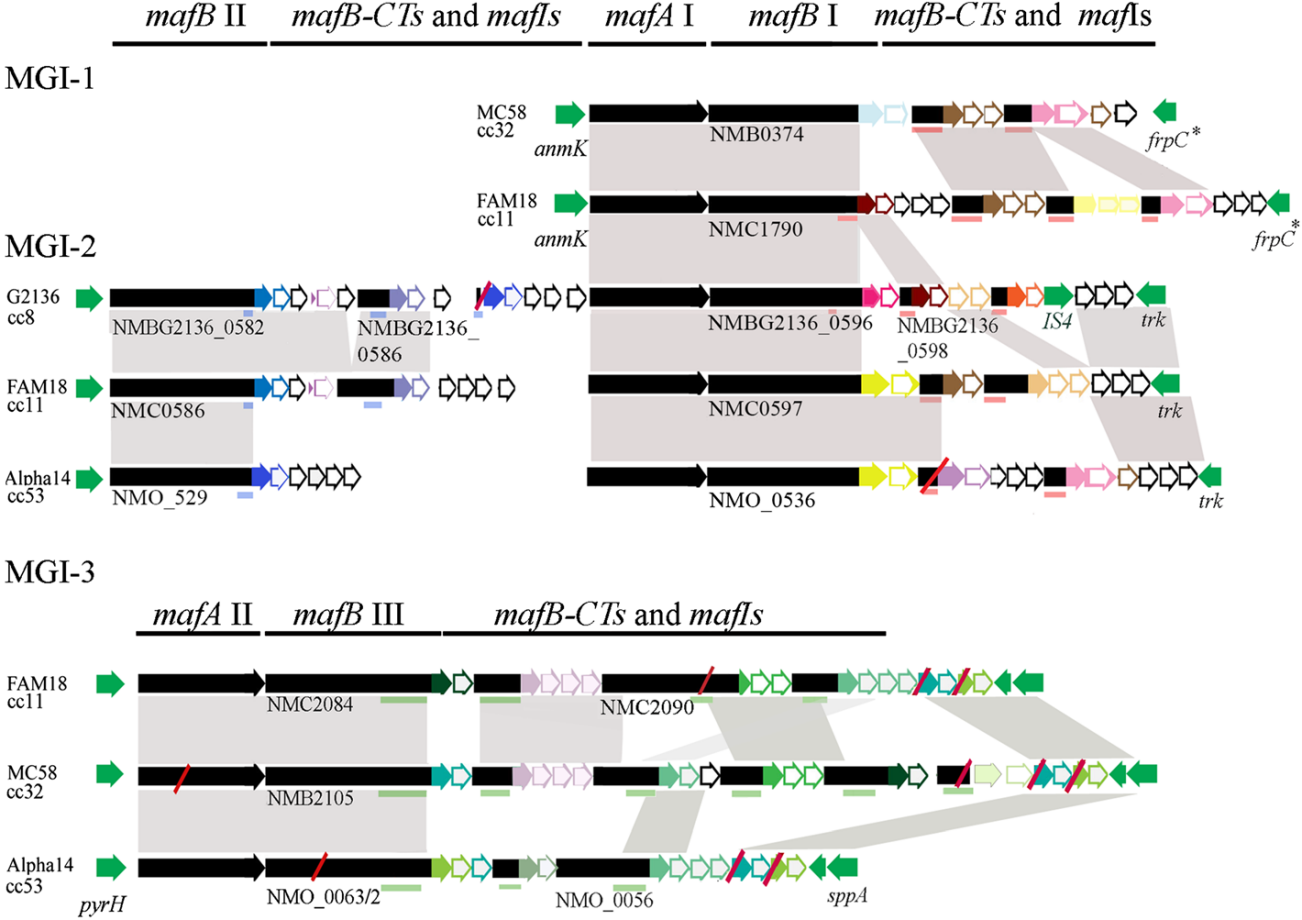
Organization of *maf* islands in various *N. meningitidis* genome sequences. Most islands contain a *mafA*, *mafB* (indicated by their locus tags) and a variable repertoire of *mafB-CT*s interspersed with ORFs, called *mafI*s, which may encode immunity proteins (indicated with open arrows). It is not clear whether all these intervenient ORFs are required to confer immunity against the toxin encoded by the upstream *mafB* or *mafB-CT* gene. Islands are classified as MGI-1, MGI-2 and MGI-3 according to [10]. The phylogenic groups of the *mafA* and *mafB* genes as defined in Fig. 2 are also indicated. The genes flanking the islands are colored green. MGI-1 contains a *mafA* and *mafB* from clusters I and is flanked by *anmk* coding for anhydro-N-acetylmuramic acid kinase and an ORF encoding a small peptide of 91 aa that shows high sequence similarity with the N terminus of the iron-regulated RTX toxin FrpC. MGI-2 contains a *mafB* of cluster II and usually also a *mafA* and *mafB* of clusters I. This island is flanked by a proline tRNA gene and *trk* encoding a potassium transport system. MGI-3 contains *mafA* and *mafB* genes of clusters II and III, respectively, and is flanked by genes encoding UMP kinase (*pyrH*) and the putative signal peptide peptidase SppA. The *mafB* and *mafB-CT* genes can share sequence identities in their central and 5’ regions, respectively (underlined), that are probably used for homologous recombination. These regions are specific for each *mafB* group, which is indicated by the different colors of the underlining. The 3’ end of the *mafB* and *mafB-CT* genes corresponds to the toxic domain; it is hypervariable as indicated by different colors. The *mafI*s remain associated with the 3’ end of the upstream *mafB* or *mafB-CT*s during genetic rearrangements in the *maf* islands; therefore, they are colored accordingly. Often additional intervenient ORFs are also found behind the *mafI*s (colored black); whether they also contribute to conferring immunity or have other functions (or no function at all) is not clear. The presence of an IS4 transposase gene in MGI-2 of strain G2136 is also indicated. Disrupted genes are indicated with red slashes. Regions that share more than 85 % of sequence similarity between two islands are indicated with grey shadows. ORFs indicated in this figure are based on genome annotations and our own analysis

**Fig. 2:**
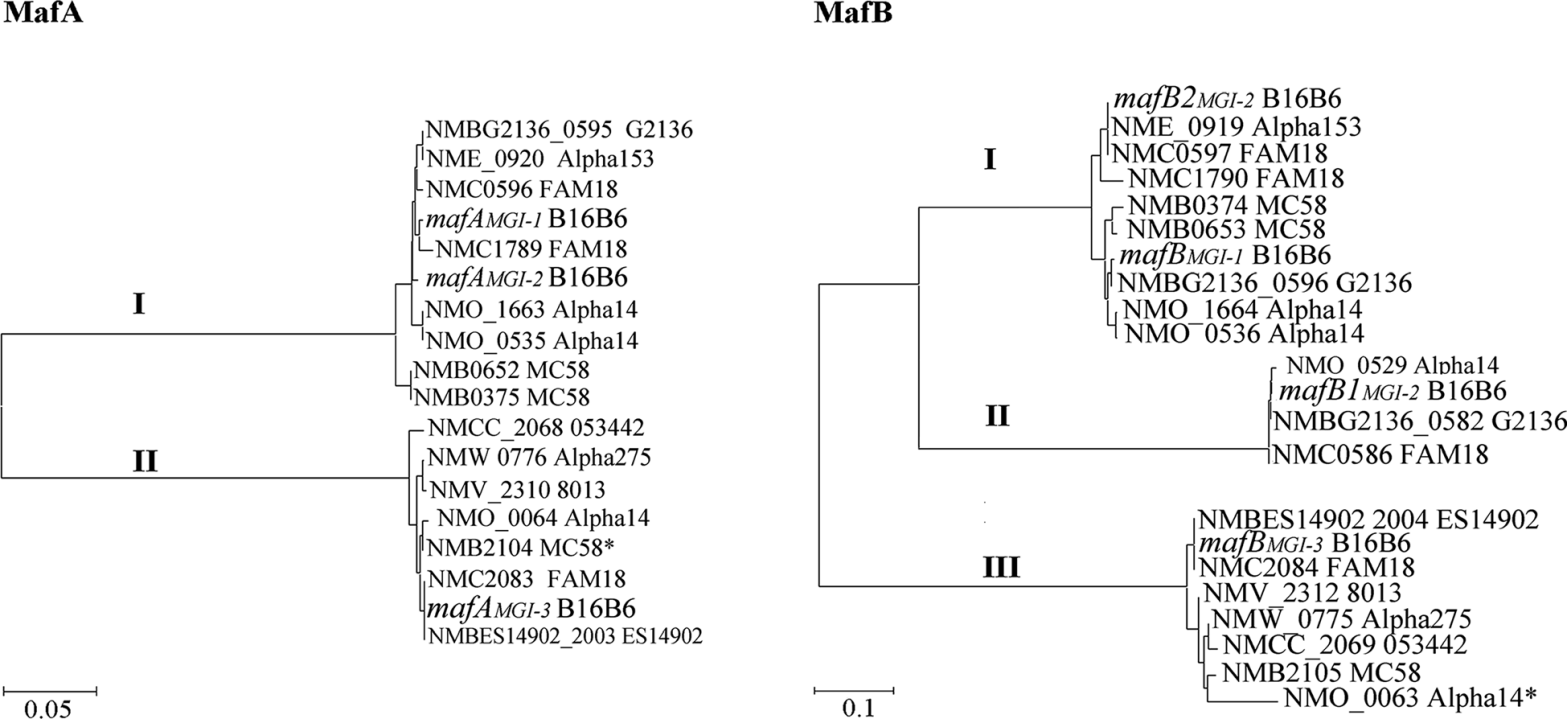
Phylogenetic trees of MafA and MafB proteins. For the construction of the trees, the amino-acid sequences of predicted mature MafA proteins and the N-terminal DUF1020 domain of predicted MafB proteins of different meningococcal strains were compared. The genetic distances are indicated with horizontal lines at the bottom

BLAST searches using different toxic domains of various meningococcal TpsA and TpsC sequences as queries yielded hits with various TpsAs and TpsCs of different bacterial species. Additional hits were also retrieved with the C termini from a large variety of other proteins, including neisserial MafB proteins. MafB proteins are present in different *Neisseria* spp., including *N. meningitidis*, *N. lactamica* and *N. gonorrhoeae*, and they were earlier assigned as members of a *multiple adhesin family* of proteins thought to be involved in adhesion to host cells [9]. The sequence similarity of MafB with TpsAs or TpsCs is restricted to the C-terminal toxic module, indicating that MafB is not secreted via a TPS mechanism. Inspection of its genetic context in available genome sequences indicated that the *mafB* genes are components of genetic islands. The genes in the islands may form an operon composed, from 5’ to 3’ end, of *mafA*, *mafB* and a variable number of *mafB*-related genes, potentially encoding alternative C-terminal domains and designated *mafB-CT*. These *mafB* and *mafB*-*CT* genes are interspersed with one or more intervenient ORFs, which may encode immunity proteins (designated *mafI*) (see Fig. 1 for examples). Up to three independent *maf* genetic islands, present on different chromosomal locations, can be recognized in meningococcal genomes (Fig. 1), designated MGI-1, 2, and 3 according to a recent proposal [10]. The predicted MafA proteins contain a lipoprotein signal sequence, and phylogenetic analysis of MafA proteins from different strains of various *Neisseria* spp. revealed clustering of the sequences in two phylogenetic groups (Fig. 2) with > 95 % of identity within each group and < 70 % identity between groups. MafB proteins contain a predicted N-terminal signal sequence, and the mature part is further structured into three regions: an N-terminal DUF1020 domain of ~ 260–320 aa in length, a central region of ~140 aa containing a Hint domain, which is usually involved in autocatalytic protein splicing, and a variable C-terminal toxic domain of ~80-100 aa. The N-terminal DUF1020 domains of MafB proteins can be clustered in three variants, I, II and III, where variants I and II are phylogenetically more related (Fig. 2). The central region is conserved in sequence, but its presence is variable as some MafBs lack the Hint domain. The C-terminal region is polymorphic and constitutes an independent toxic domain. The *mafB-CT*s are variable in length (260–893 bp). At their 3’ end, they contain sequences that putatively encode a toxic domain, whilst upstream thereof they share sequence similarity with sequences adjacent to the toxic region of *mafB* genes, including sometimes an intact Hint domain. Some *mafB-CT*s, for instance NMBG2136_0586 of strain G2136 (Fig. 1), encode a VENN domain previously recognized as being adjacent to the toxin domain in TpsA proteins [2]. The *mafB-CT* genes generally lack sequences corresponding to the 5´ end of *mafB* genes, but occasionally, a complete *mafB* is found among the *mafB-CT* genes, for example NMO_0056 or NMC2090 in MGI-3 of strains Alpha14 and FAM18, respectively (Fig. 1). The shared sequences between *mafB* and *mafB-CT* genes may be used for genetic rearrangements, resulting in the substitution of the toxic domain at the C terminus of MafB. Examination of various neisserial genome sequences suggests that such recombination events have indeed occurred in several cases, since the C-terminal domains of some MafB proteins have > 95 % of sequence similarity with predicted MafB-CT proteins from other genomes. For example, the 3’ end of *mafB-CT* NMBG2136_0598 in MGI-2 of G2136 is placed at the 3’ end of the *mafB* NMC1790 in MGI-1 of strain FAM18 (Fig. 1). In general, such rearrangements also affect the position of the immediately downstream located *mafI* gene, which remains associated with the 3’ end of the upstream *mafB* or *mafB-CT* gene, supporting the notion that these modules function as specific toxin-antitoxin pairs. However, in some islands, several *mafI*s are associated with a specific *mafB* or *mafB-CT* (Fig. 1); it is not clear whether they are all required to confer immunity against the toxic domain of the corresponding MafB or MafB-CT.

Examination of the genomic islands revealed that *mafA*s of phylogenetic group I are always immediately followed by a *mafB* of group I, whilst islands containing a *mafA* of group II always contain an adjacent *mafB* of group III (Fig. 1). Remarkably, the *mafB* genes of group II (NMBG2136_0582, NMC0586 and NMO_0529 of strains G2136, FAM18, and Alpha14, respectively, in Fig. 1) are not directly adjacent to a *mafA*, but they are located on an island (MGI-2) usually also containing a *mafA* of group I and a *mafB* of group I. Furthermore, the *mafB-CT*s only share sequences with the upstream located *mafB* or *mafB*s of the same phylogenetic group located on a different island (see Fig. 1 for examples), suggesting the existence of independent *mafB/mafB-CT* systems with genetic exchange between *mafB* and *mafB-CT*s being restricted to each system.

Overall, this analysis revealed that Maf systems share several genetic features with the TPS systems involved in interbacterial competition. Accordingly, it is logical to hypothesize that (i) MafB is a secreted protein with growth-inhibitory properties, (ii) MafI proteins confer specific immunity against the toxic MafB, and (iii) *mafB-CT*s represents a source of switchable toxic modules.

### Characterization of MGIs in strain B16B6

**Fig. 3:**
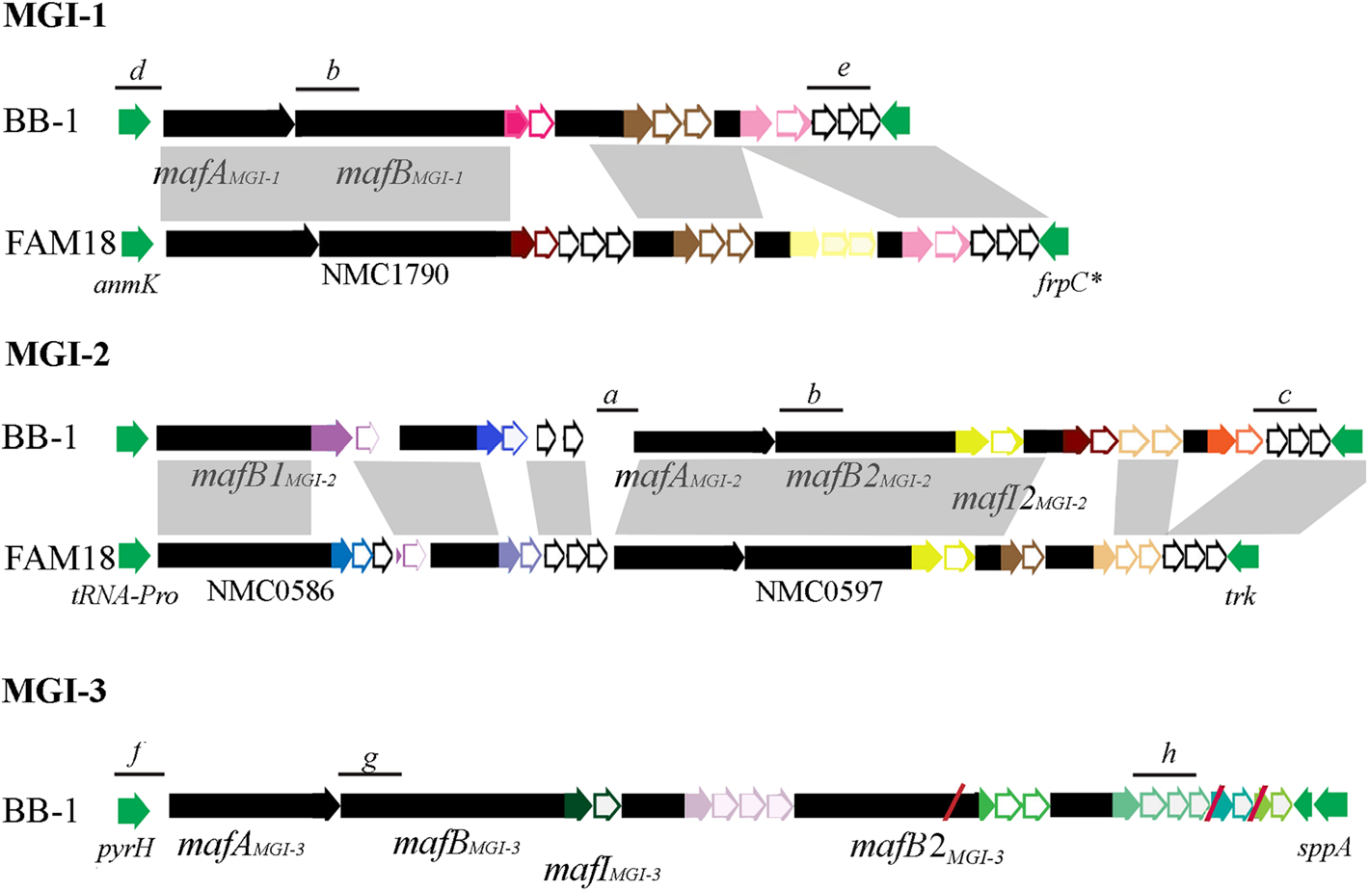
Genetic organization of the MGI of strain B16B6. B16B6 contains three MGIs. The genetic composition of each island and their flanking regions (indicated by solid green arrows) are in accordance with those observed in other genomes (Fig. 1); therefore, the same classification is applied. The different genomic features of each island are illustrated as indicated in the legend to Fig. 1. Color coding of genes is also similar as in Fig. 1. The organization of the MGI of strain B16B6 is compared with those of FAM18, which is of the same clonal complex cc11; similarities are indicated with grey shadowing as in Fig. 1. MGI-3 of FAM18 is similar to that of B16B6 and is therefore not depicted. DNA fragments targeted by PCR for the generation of knockout constructs are indicated by lines and numbered a-h; the corresponding primers are presented in Additional file 1: Table S1

To investigate our hypotheses, we used *N. meningitidis* strain BB-1, an unencapsulated derivative of B16B6 of the clonal complex cc11 [11], in which TpsA-mediated growth-inhibitory activity was previously demonstrated [4, 12]. Extensive PCR analysis and subsequent sequencing of PCR fragments revealed that this strain contains three MGI corresponding with islands MGI-1 (GenBank accession number KR006907), MGI-2 (GenBank accession number KR012999), and MGI-3 (GenBank accession number KR006906) in other meningococcal genomes (Fig. 3). Comparison of the genetic organization of the MGIs of strains FAM18 and B16B6, which are both of the same clonal complex (cc11), revealed considerable differences (Fig. 3). The genetic composition of MGI-3 is similar in both strains, with > 99 % of sequence identity. However, comparison of islands MGI-1 and MGI-2 of both strains revealed complex rearrangements. In MGI-2, the toxic domain encoded by the first *mafB* gene (*mafB1*_*MGI-2*_) and the downstream *mafI* and *mafC* genes differ between the two strains (Fig. 3). In contrast, the second *mafB* gene in this island (*mafB2*_MGI-2_) contains a similar 3’ end in the two strains. However, downstream of *mafB2*_MGI-2_ and its cognate *mafI*, there are large differences in the *mafB-CT* and *mafI* content of the two strains (Fig. 3), and the organization in this part of island MGI-2 of strain B16B6 is actually very similar to that of MGI-2 of cc8 strain G2136 (Fig. 1), except that the IS4 transposase gene is lacking. In island MGI-1, the 3’ end of the *mafB* of B16B6 is different from that of FAM18 (Fig. 3) but similar to that of the second *mafB* in MGI-2 of strain G2136 (locus tag NMBG2136_0596; Fig. 1). After the cognate *mafI*, the organization of *mafB-CT* and *mafI* genes in MGI-1 of B16B6 is also deviant from that in FAM18 (Fig. 3) but similar to that in the corresponding island of strain MC58 (compare Figs 1 and 3).

The very different organization of the MGI-1 and MGI-2 islands in cc11 strains B16B6 and FAM18 is striking, since comparative analysis of the TPS island in these strains and a large collection of other cc11 strains evidenced only a very low frequency of genetic rearrangements [4]. To confirm the genetic variability of the *maf* loci, we extended our analysis to available genome sequences of nine other cc11 strains, i.e. ES14902, M6190, M7124, NM3682, NM3683, NM126, M10208, NM174 and NM82. In all these strains, the C terminal toxic domains of MafB2_MGI-2_ and MafB_MGI-3_ are similar to those of FAM18 and B16B6, whilst that of MafB_MGI-1_ is similar to that in B16B6 but different from that in FAM18. In two strains, *i.e.* NM3682 and NM3683, the toxic domain of MafB1_MGI-2_ is similar to that of B16B6, while it is similar to that of FAM18 in the other seven strains. Thus, these results suggest that the C-terminal toxic domain of MafB of phylogenetic cluster III, which is encoded by MGI-3, is rather stable in strains of the same cc11 lineage, as previously found for TpsA [4], but the MafBs of clusters I and II, which are encoded by MGI-1 and MGI-2, seems to be more prone to alteration.

### MafB functions in meningococcal growth inhibition

**Fig. 4:**
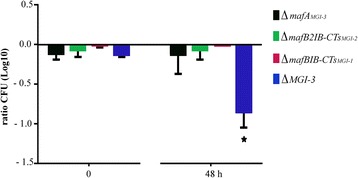
Growth inhibition assays. Cells of a rifampicin-resistant derivative of BB-1 were mixed 1:1 with cells of mutant strains Δ*mafA*_*MGI-3*_, lacking *mafA* of MGI-3, Δ*mafB2IB-CTs*_*MGI-2*_, lacking *mafB2* and all downstream genes of MGI-2, Δ*mafBIB-CTs*_*MGI-1*_, lacking *mafB* and all downstream genes of MGI-1, or ΔMGI-3,lacking the entire MGI-3 island, all carrying a kanamycin-resistance cassette. The suspensions were spotted on GC plates without antibiotics and incubated for 48 h. The ratios of the mutants over wild-type bacteria in the spots were determined by plating on GC media containing kanamycin or rifampicin and counting colony-forming units after overnight incubation. Results are means and s.d. of three independent experiments. Statistically significant differences between time 0 and 48 h are marked with an asterisk (unpaired *t*-test of *P* < 0.001). All strains tested here did not show differences in viability when grown separately

To investigate the putative role of MafB proteins in mediating growth inhibition, we first constructed several mutants of strain BB-1, lacking diverse genes in different *maf* islands. These mutants were mixed with a rifampicin-resistant derivative of the parent strain in different ratios and the numbers of wild-type and mutant bacteria were determined by plating on selective media at time 0 and after different periods (3 –5 h) of co-incubation at 37 °C while shaking. However, under these conditions, we did not observe inhibition of the growth of the mutants relative to the parent. Therefore, we next performed these assays on GC plates in a candle jar, conditions that were previously found to be optimal to detect TpsA-mediated growth inhibition [4]. Under these conditions, growth of a mutant lacking the complete MGI-3 island (ΔMGI-3; indicated in blue in Fig. 4) and of a mutant lacking the entire MGI-3 island except for *mafA* (data not shown) were significantly reduced in the presence of the parent, whilst that of a mutant lacking only *mafA* of MGI-3 was not inhibited (Δ*mafA*_*MGI-3*_; indicated in black in Fig. 4). When a mutant lacking *mafB2* and all downstream genes of island MGI-2 (Δ*mafB2IB-CTs*_*MGi-2*_; green in Fig. 4), or a mutant lacking all genes except for *mafA* of island MGI-1 (Δ*mafBIB-CTs*_*MGI-1*_; red in Fig. 4), were tested in similar conditions, no significant growth inhibition by the parent was detected. These results demonstrate that at least island MGI-3 mediates growth inhibitory activities.

### *mafB* and *mafI* genes encode toxins and cognate immunity proteins

**Fig. 5:**
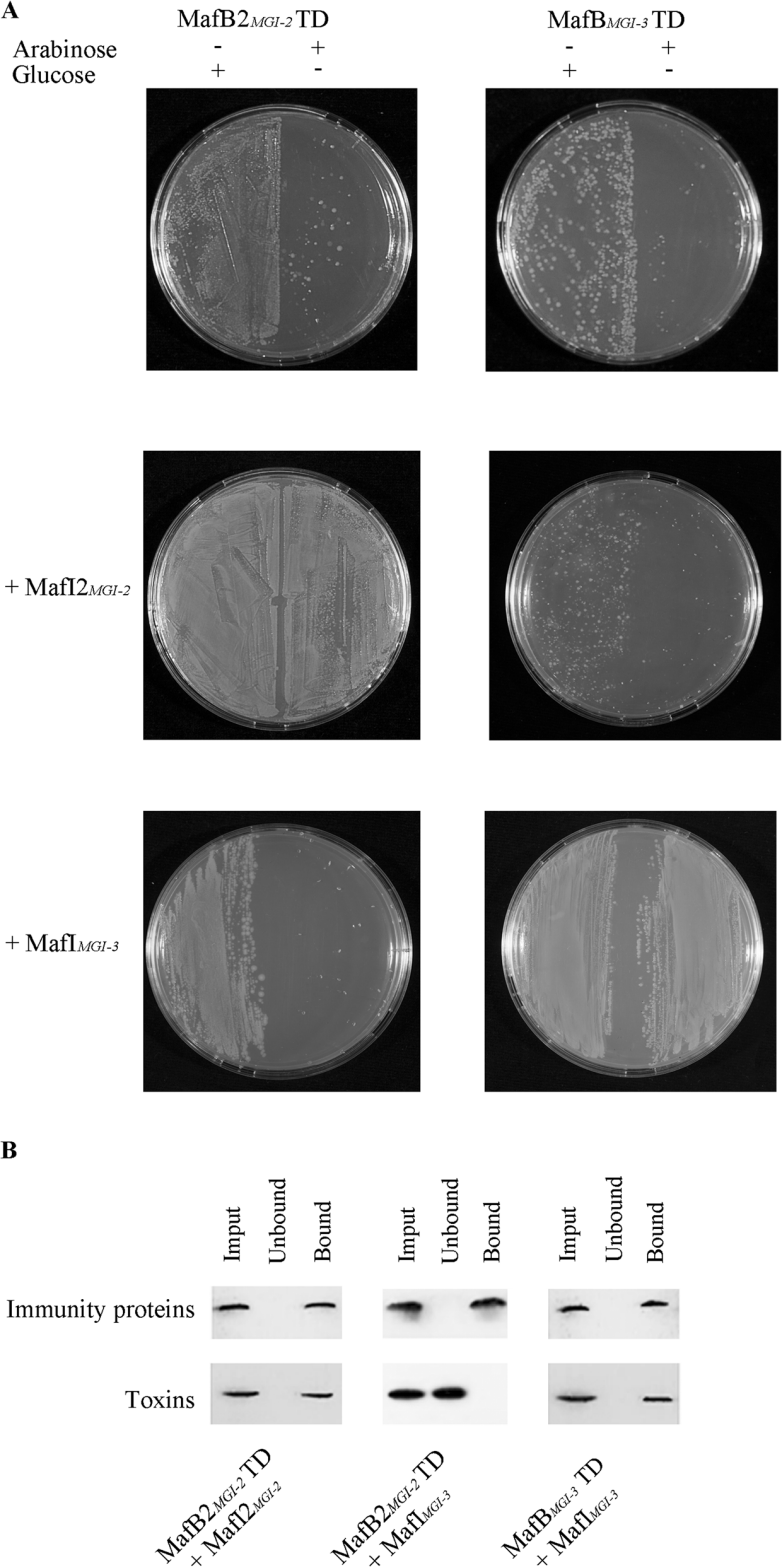
*MafB* and *mafI* genes code for toxins and cognate immunity proteins, respectively. **a** The recombinant C-terminal, putatively toxic domains (TD) of MafB2_MGI-2_ and MafB_MGI-3_ and the immunity proteins MafI2_MGI-2_ or MafI_MGI-3_ of strain B16B6 were expressed in *E. coli* strain BL21 (DE3) and survival was determined. Expression of the MafB TD fragments was induced or repressed with arabinose or glucose, respectively, as indicated on top of the figure and expression of the *mafI* genes was induced with IPTG when the corresponding plasmids were present. After overnight incubation, 20 μl of the bacterial suspensions were spread on half of an LB agar plate containing appropriate antibiotics, and the plates were incubated overnight at 37 °C. **b** MafI specifically interacts with the C-terminal TD of the cognate MafB. BL21 (DE3) cells co-expressing a Flag-tagged C-terminal MafB TD and a His-tagged MafI were disrupted by sonication, and the lysate was mixed with Ni^2+^ NTA resin. Aliquots of resin-bound and unbound fractions were analyzed by Western blotting with anti-Flag and anti-His monoclonal antibodies

To investigate whether indeed the C-terminal part of MafB_MGI-3_ is responsible for the growth-inhibitory effect observed in the *in vivo* assays, we cloned the DNA for this fragment from strain B16B6 with a FLAG-epitope tag into a pAYC184-derived vector behind an arabinose-inducible promoter. Although we did not detect growth inhibitory activity *in vivo* of the MafBs of phylogenetic cluster I encoded by islands MGI-1 and MGI-2, we decided to clone also, as a representative, the DNA for the C-terminal fragment of MafB2_MGI-2_ to investigate whether also this protein is a toxin. It is noteworthy that this domain is recognized in the Conserved Domain Database (CDD) as a toxin64 domain with a possible RNase function. In contrast, the C-terminal domain of MafB_MGI-3_ of B16B6 was not recognized in CDD as a putative toxic domain, but it was predicted to be a toxin_56 (putative RNase) with 95 % probability using the HHpred server. We also cloned the *mafI*s located immediately downstream of these *mafB*s on the chromosome into a pET26b vector behind an isopropyl-β-D-1-thiogalactopyranoside (IPTG)-inducible T7 promoter; the resulting recombinant proteins contain a His_6_ tag on their C-terminal end. The plasmids were introduced in *E. coli* strain BL21 (DE3). The expression of the recombinant proteins was independently controlled with specific inducers and tested by Western blotting, and cell viability after overnight induction was tested by plating the bacterial suspensions on LB plates with appropriate antibiotics. The expression of the C-terminal fragment of MafB2_MGI-2_ in *E. coli* resulted in a drastic reduction of cell viability compared to the non-induced control (Fig. 5a). Viability was restored when the cognate MafI2_MGI-2_ was co-expressed, but not by co-expression of MafI_MGI-3_. Similarly, the expression of the C-terminal part of MafB_MGI-3_ resulted in a drastic reduction of cell viability, which could be restored by co-expression of the cognate MafI_MGI-3_ but not of the non-cognate MafI2_MGI-2_ (Fig. 5a). These assays confirmed that indeed the C-terminal parts of both MafBs are toxic and that their activity is specifically inhibited by the cognate MafI.

Usually, the immunity proteins of toxin-antitoxin systems confer protection through their direct interaction with the cognate toxins. To investigate whether the MafI proteins interact with the C-terminal fragments of the corresponding MafBs, pull-down assays were performed. The His-tagged immunity proteins were purified on Ni^2+^ beads from *E. coli* cells also expressing the C-terminal MafB fragments, and the co-purification of the MafB fragments was examined on Western blots. As anticipated, both MafB2_MGI-2_ and MafB_MGI-3_ C-terminal fragments were purified together with their cognate immunity proteins but not with non-cognate MafI (Fig. 5b). Thus, these assays confirmed the role of MafI in conferring protection by specifically interacting with C-terminal domain of the cognate MafB.

## Discussion

Toxins play an important role in the competition between bacteria for limited resources. They are produced to inhibit the growth of competitors for the same niche and, in addition, they are used for self recognition in a bacterial community. Bacteria have developed several independent competition systems, including bacteriocins, TPS systems, type 6 secretion systems, Rhs, or the ESX systems in Gram-positive bacteria [13–15]. The neisserial Maf systems appear to share features with several of these previously characterized competition systems. Growth inhibition in these systems is mediated by a cell-surface-exposed or secreted protein, *i.e.* MafB in the case of the Maf system. Like in many other systems, the C-terminal end of MafB contains a polymorphic toxic module, whilst the rest of the protein might be required for its secretion and/or for its interaction with receptors on the target cells. Producing cells are protected against the toxic activity by a small protein, *i.e.* MafI in the Maf system, which interacts with the toxin and is usually encoded by the gene immediately adjacent to the toxin gene. Like in several other systems, the *mafB* and *mafI* genes are located on genetic islands together with a variable repertoire of genes putatively encoding N-terminally truncated toxins, called *mafB-CT*s in the Maf system [10], with the cognate immunity genes. By genetic rearrangements, these *mafB-CT*s may substitute the 3’ end of the *mafB* gene, thereby replacing its toxic activity, whilst the array of encoded immunity proteins may confer protection against a variety of toxins that may be encountered in the natural habitat. Indeed, a variety of toxic domains may be encoded at the 3’ end of the same *mafB* gene in different genomes, and the same domains may be found at the 3’ end of *mafB-CT* genes in other genomes (Fig. 1), clearly supporting the idea that such rearrangements occur *in vivo*. The substitution of the toxic module may offer a selective advantage in a given bacterial community [4, 16] but may also lead to the loss of immunity genes [4]. Indeed, an analysis of the frequency of substitution of toxic domains in the *tpsA* loci of *N. meningitidis* evidenced that this event occurs but is very rare in isolates of the same genetic lineage and that such alteration may generate a competitive disadvantage relative to other members of the community [4]. Similarly, our analysis of a limited set of strains of the same genetic lineage in the current study evidenced no alterations at the C terminus of MafB proteins of phylogenetic group III encoded on MGI-3. However, rearrangements were evident in MafB proteins of phylogenetic groups I and II encoded on MGI-1 and MGI-2. Note that the mutual similarity between MafB proteins of groups I and II is higher than their similarity to group 3 (Fig. 2). It is conceivable that MafB_MGI-3_ plays a role in the maintenance of the bacterial community, while MafB proteins encoded on MGI-1 and MGI-2 may offer a selective advantage to members of a community only under specific conditions. Perhaps, this may explain why we detected growth inhibitory activity only of MafB_MGI-3_ and not of MafB_MGI-1_ and MafB2_MGI-2_. Possibly, the latter MafB proteins or their cognate receptors on the target cells are not expressed under the experimental conditions used. Alternatively, these proteins may target other bacterial species or eukaryotic cells. This question remains to be elucidated in future work.

The secretion mechanism of MafB was not elucidated in this work. MafB proteins were detected in the culture supernatant of *N. meningitidis* strain when overexpressed from a plasmid but the secretion mechanism was not solved [10]. MafB proteins are synthesized with an N-terminal signal sequence for transport across the inner membrane via the Sec system. Furthermore, all mature MafB proteins contain a conserved DUF2010 domain at their N-terminal end, which may constitute a recognition signal for the machinery that mediates its subsequent transport across the outer membrane. The MafA proteins, previously reported to function as glycolipid-binding adhesins [17], are likely candidates to constitute this machinery. Interestingly, our phylogenetic analysis revealed two main clusters of MafA variants, which paralleled the clustering of the MafB proteins into two main clusters based on their DUF1020 domains, suggesting specificity in the recognition of MafB proteins by the cognate MafA. However, MafA is a predicted lipoprotein and secondary structure predictions were inconsistent with a β-barrel conformation, which is common among integral outer membrane proteins, including, for example, the TpsB proteins, which mediate the transport of TpsA proteins across the outer membrane in the TPS systems [1]. Perhaps, the quaternary structure of MafA generates a channel in the outer membrane as reported, for example, for the CsgG protein, which mediates the translocation of curli subunits across the outer membrane [18].

Some MafB proteins contain a Hint domain in between the DUF1020 domain and the toxic domain. Such domains are responsible and sufficient for protein splicing and could have a role in toxin processing and delivery into the target cells. There are two types of Hint domains, designated type A and B, based on sequence motifs [19]. The Hint domains, if present in MafB proteins, are of type A. Proteins containing this domain undergo splicing and release of the C-terminal part [19]. However, most MafB proteins do not contain such a Hint domain; how the C-terminal toxic moiety is released and transported into the target cells in those cases is not clear and remains to be investigated.

Whilst this manuscript was in preparation, an independent study reported the toxic activity of the C-terminal domains of some of the MafB proteins of another meningococcal strain, 8013, and the competitive advantage of overexpression of one of these MafB proteins [10], consistent with the observations reported in this paper.

## Conclusions

In this work, we identified the neisserial MafB proteins as a novel family of toxic proteins involved in interbacterial competition. We demonstrated that the toxic activity of MafB of strain B16B6 resides in the C-terminal region, while MafI specifically interacts with this toxic domain to confer protection to the producing cells. Further studies will be directed to elucidate the secretion mechanism of MafB and to identify possible applications of this system to fight pathogens.

## Methods

### Bioinformatics analysis

The C-terminal 150 aa of several meningococcal TpsA and TpsC proteins present in available genome sequences were used as input queries in BLAST searches. Analysis and comparisons of DNA and protein sequences were performed with Clone Manager software Suite 7. Alignment of protein sequences was performed in MAFFT version 7 (http://mafft.cbrc.jp/alignment/server/). Aligned sequences were then used for phylogenetic analyses using the Neighbour-joining method with the available MEGA software version 6 (http://www.megasoftware.net/). The matrices and phylogenetic trees derived from this study were deposited in Treebase repository (http://treebase.org/treebase-web/search/study/anyObjectAsRDF.rdf?namespacedGUID=TB1:S17890). The subcellular localization of proteins was predicted using the servers SignalP (http://www.cbs.dtu.dk/services/SignalP/) and LipoP (http://www.cbs.dtu.dk/services/LipoP/). Secondary and tertiary structure of the mature proteins were predicted using the public web-based programs Phyre2 (http://www.sbg.bio.ic.ac.uk/phyre2/html/page.cgi?id=index) and PSIPRED v3.3 (http://bioinf.cs.ucl.ac.uk/psipred/). For homology detection, the HHpred server was used at http://toolkit.tuebingen.mpg.de/hhpred.

### Bacterial strains and growth conditions

*N. meningitidis* strain BB-1 is a non-capsulated derivative of strain B16B6 of clonal complex cc11 [11]. A spontaneous rifampicin-resistant mutant of BB-1 was also described [11]. The bacteria were grown at 37 °C on GC medium base (Difco) supplemented with IsovitaleX (Becton Dickinson) and appropriate antibiotics (100 μg ml^−1^ of kanamycin, 5 μg ml^−1^ of chloramphenicol, or 50 μg ml^−1^ of rifampicin) at 37 °C in a candle jar. For liquid cultures, bacteria grown on plates were diluted in tryptic soy broth (TSB; Beckton Dickinson) to an OD_550_ of 0.1 and incubated in 25-cm^2^ polystyrene cell-culture flasks with constant shaking at 110 rpm.

*E. coli* strains DH5α and BL21 (DE3) (Invitrogen) were grown in lysogeny broth (LB) or LB agar at 37 °C. When required, the media were supplemented with antibiotics (100 μg ml^−1^ of kanamycin, 25 μg ml^−1^ of chloramphenicol, or 10 μg ml^−1^ of tetracycline).

### PCR amplification and sequencing

Chromosomal DNA was extracted from BB-1 and its mutant derivatives as described [4] and used as template DNA for PCRs. PCRs were performed using 1–2 μl of extracted DNA, 200 μM dNTPs (Fermentas), 0.25 μM of different primer combinations (see in Additional file 1: Table S1), 0.5 U of Expand High Fidelity Enzyme Mix, and PCR buffer of the Expand High Fidelity PCR System (Roche). Thermal cycling conditions were 30 cycles of 1 min at 95 °C, 0.5 min at 58 °C and elongation at 72 °C during 1 min per kbp of expected amplicon size. Reactions were preceded by incubation at 95 °C for 10 min and terminated with an extended elongation step for 10 min at 72 °C. The PCR products were separated by electrophoresis in 1 % agarose gels and stained with ethidium bromide for visualization. Some PCR products were cloned into the pCR2.1-TOPO vector (Invitrogen), according to the manufacturer’s instructions. Plasmids and PCR products were purified using the Plasmid Extraction kit and Clean-Up System, respectively, (Promega). Purified plasmids and PCR products of variable length were sequenced at the Macrogen sequencing service (Seoul, Korea). For sequencing the *maf* islands, several overlapping DNA fragments were amplified by PCR, and then sequenced either directly or after cloning in the TOPO vector using internal primers. Amplification reactions were carried out independently and repeated at least twice for each fragment. Assembly of sequences was performed with SeqMan II software (DNAstart Inc.).

### DNA manipulation techniques

Cloning was performed in *E. coli* DH5α. All primers used for PCR amplifications with the restriction sites they contain are listed in Additional file 1: Table S1. To generate knockout constructs, DNA fragments upstream and downstream of the gene (s) of interest were amplified from chromosomal DNA of strain B16B6 as indicated in Fig. 3. PCR products were purified, digested with proper restriction enzymes, and sequentially cloned into p*nhbA*::*kan* [4] thereby replacing DNA fragments flanking the kanamycin-resistance cassette in the original plasmid. The knockout constructs were purified and used to transform strain B16B6. Appropriate PCRs were used to verify the presence of the correct mutations in kanamycin-resistant transformants.

For expression of recombinant proteins in *E. coli*, DNA fragments corresponding to the toxic regions (the C-terminal 100 aa residues) of the MafB proteins MafB2_MGI-2_ and MafB_MGI-3_ and the cognate MafI proteins of BB-1 were amplified by PCR. The *mafI* genes were cloned into the expression vector pET26b behind a T7 promoter, which is inducible with IPTG and repressed with glucose. Cloning in this vector resulted in a 6xHis-tag at the C terminus of the recombinant protein. The *mafB* gene fragments were cloned behind the arabinose-inducible *araBAD* promoter (*P*_*BAD*_) on a pACYC184-derived vector. This vector was prepared by amplifying *P*_*BAD*_ together with the downstream *yciM*-*phoA* chimeric gene from plasmid pBAD*yciM*-*phoA* [20] and cloning the resulting PCR product into the chloramphenicol-resistance gene on pACYC184 [21], resulting in plasmid pACYC184-*P*_*BAD*_*-yciM-phoA*. The *mafB* fragments were substituted for the *yciM-phoA* chimeric gene on pACYC184-*P*_*BAD*_*-yciM-phoA*. The cloning procedure resulted in the presence of an N-terminal FLAG tag in the recombinant proteins. To avoid the lethal effect of the production of the toxic MafB fragments, the plasmids encoding these fragments were introduced in DH5α and BL21 (DE3) under repression conditions (*i.e*. in the absence of arabinose and the presence of 2 % glucose), and when necessary, the expression of the cognate MafI from a plasmid in *trans* was induced. The correct expression of recombinant proteins was determined by Western blotting.

### Growth inhibition assays

For growth inhibition assays, the rifampicin-resistant derivative of BB-1 was used as the killer and various *maf* mutant derivatives of BB-1 containing a kanamycin-resistance marker as target cells. Growth inhibition was performed as described [4, 12]. Briefly, bacteria from overnight cultures were grown independently in TSB for 4–5 h. Strains were then mixed 1:1 in TSB and drops were spotted on GC medium plates and incubated for 48 h at 37 °C in candle jars in a humid atmosphere. Subsequently, bacteria were scraped from the plates, resuspended in TSB, and the ratio of the different bacteria at time 0 and after 48 h of co-incubation was determined by plating on selective GC plates containing either rifampicin or kanamycin and counting colonies after overnight incubation. Data of three independent experiments performed in duplicate were considered for statistical comparisons using an unpaired statistical *t*-test.

### Toxicity in *E. coli* and pull-down assays

*E. coli* BL21 (DE3) harboring plasmids carrying the 3’ end of *mafB* and *mafI* genes were used for toxicity assays. Bacteria from overnight cultures were diluted to an OD_600_ of 0.1 in 10 ml of LB with appropriate antibiotics for plasmid selection. The concentrations of glucose (0.5 or 2 %), arabinose (2 %) and IPTG (0.1 or 1 mM) used for repression or induction of protein synthesis were optimized for each strain. After overnight incubation, bacteria were plated on LB plates containing proper antibiotics for determination of cell viability. The assays were repeated at least three times. Plates of a representative experiment were photographed with a conventional camera.

For pull-down assays, recombinant MafB and MafI proteins were simultaneously overproduced and the His-tagged MafI was purified by affinity chromatography using Ni^2+^-NTA beads (QIAGEN GmbH, Hilden) according to the manufacturer’s instructions. Briefly, bacteria were lysed by sonication in 50 mM sodium phosphate, pH 8.0, 300 mM sodium chloride, and 10 mM imidazole, and cell debris was removed by centrifugation (6000 *g*, 30 min). The supernatant was mixed with Ni^2+^ beads, and the beads were washed with 50 mM sodium phosphate, pH 8.0, 300 mM sodium chloride, 20 mM imidazole, and eluted with the same buffer containing 250 mM imidazole. The correct purification of the His-tagged MafI and the co-purification of the FLAG-tagged MafB were examined by SDS-PAGE and Western blotting.

### Sample preparation, SDS-PAGE and Western blotting

For sample preparation, cultures were adjusted to an OD_600_ of 1.0, and cells were collected by centrifugation (8000 rpm during 3 min, Eppendorf Centrifuge 5424) and suspended in sample buffer. Purified proteins eluted from Ni^2+^ beads were mixed with double-strength sample buffer. SDS-PAGE was performed at 200 V during ∼ 45 min in a discontinuous buffer system using 12 % or 15 % polyacrylamide gels. Gels were stained with Coomassie brilliant blue G250 or the proteins were transferred to nitrocellulose membranes. Membranes were subsequently blocked with PBS containing 0.1 % (v/v) Tween 20 (PBST) and 0.5 % (w/v) non-fat dried milk (PBST-M) for 1 h and incubated with anti-His (Thermo Fisher Scientific) or anti-FLAG monoclonal antibodies in PBST-M for 1 h at room temperature. After washes with PBST, the membrane was incubated for 1 h with horseradish peroxidase-conjugated goat anti-mouse immunoglobulins diluted in PBST-M. After extensive washing in PBST, blots were developed with the Pierce ECL Western blotting substrate.

## Additional file

Additional file 1: Table S1. Primers used in this study. (PDF 256 kb)

## Abbreviations

Aa: Amino acids
bp: Base pairs
cc: Clonal complex
IPTG: Isopropyl-β-D-1-thiogalactopyranoside
kbp: Kilobase pairs
LB: Lysogeny broth
PBST: PBS containing 0.1 % (v/v) Tween 20
PBST-M: PBST with 0.5 % (w/v) non-fat dried milk
PCR: Polymerase chain reaction
TPS: Two-Partner Secretion
TpsA: Two-partner secretion protein A
TSB: Tryptic soy broth
rhs: Rearrangement hot spot
