# Supplementary material for: Fratricide activity of MafB protein of N. meningitidis strain B16B6
Source: BMC Microbiol. 2015 Aug 5;15:156. doi: 10.1186/s12866-015-0493-6 (PMC4524018; doi:10.1186/s12866-015-0493-6)
Supplement: Additional file 1: Table S1. — Primers used in this study. (PDF 256 kb) [file 12866_2015_493_MOESM1_ESM.pdf]

Table S1. Primers used in this study

Goal PCR	Fragment[a]	Primer name[b]	Sequence (5'->3')[c]	Remarks
Preparation of knock out constructs				
Δ*mafA*$_{MGI-2}$	*a*	FwNMC0595	CGCGCGAGATCTTGGAAAATTACGAAAGAGCAAGGTCAAGATTTA	BglII
		RvNMC0595	CGCGCGCCATGGGGCGGGTATGCCGGTCAGTGTGCCGCA	NcoI
	b	FwNMC1790N	CGCGCGCATATGACCGTGAAACCGCTGCGAAGACTG	NdeI
		RvNMC1790E	CGCGCGGATATCAAAAGCGGACGGTGTAACCAA	EcoRV
Δ*mafB2IB-CTs*$_{MGI-2}$	*b*	FwNMC1790B	CGCGCGAGATCTACCGTGAAACCGCTGCGAAGACTG	BglII
		RvNMC1790Nc	CGCGCGCCATGGAAAAGCGGACGGTGTAACCAA	NcoI
	c	FwNMC0602	CGCGCGCATATGATGATGAGTGTAAAAGAATTATTC	NdeI
		RvNMC0603	CGCGCGGATATCTCAACTATAATCATTTTTTCTTAAAG	EcoRV
Δ*mafA*$_{MGI-1}$	*d*	FwNMC1788	CGCGCGAGATCTATGATGGAAACACAGCTTTACATC	BglII
		RvNMC1788	CGCGCGCCATGGGGCGGGTATGCCGGTCAGTGTGCCGCA	NcoI
	b	FwNMC1790N	CGCGCGCATATGACCGTGAAACCGCTGCGAAGACTG	NdeI
		RvNMC1790E	CGCGCGGATATCAAAAGCGGACGGTGTAACCAA	EcoRV
Δ*mafBIB-CTs*$_{MGI-1}$	*b*	FwNMC1790B	CGCGCGAGATCTACCGTGAAACCGCTGCGAAGACTG	BglII
		RvNMC1790Nc	CGCGCGCCATGGAAAAGCGGACGGTGTAACCAA	NcoI
	e	FwNMC1790N	CGCGCGCATATGACCGTGAAACCGCTGCGAAGACTG	NdeI
		RvNMC1790E	CGCGCGGATATCAAAAGCGGACGGTGTAACCAA	EcoRV
Δ*mafA*$_{MGI-3}$	*f*	FwNMC2082	CGCGCGAGATCTATGGATCGCGCCACCGCCGACTACATGGGCATGATG	BglII
		RvNMC2082	CGCGCGCCATGGAGATTTTCTCCTTTGATGAAAAAC	NcoI
	g	FwNMC2083N	CGCGCGCATATGCGACACTGCCTTTCTTTCCCACTT	NdeI
		RvNMC2084E	CGCGCGGATATCGAGAAATCGACCATTAATCCTTCC	EcoRV
Δ*MGI-3*	*f*	FwNMC2082	CGCGCGAGATCTATGGATCGCGCCACCGCCGACTACATGGGCATGATG	BglII
		RvNMC2082	CGCGCGCCATGGAGATTTTCTCCTTTGATGAAAAAC	NcoI
	h	FwNMC2095N	CGCGCGCATATGGGTTACCTATCAAATAATTTACCT	NdeI
		RvNMC2095E	CGCGCGGATATCAGTTTTTTAATGACATCGGTCTAT	EcoRV
Δ*mafBIB-CTs* $_{MGI-3}$	*g*	FwNMC2082B	CGCGCGAGATCTATGGATCGCGCCACCGCCGACTACATGGGCATGATG	BglII
		RvNMC2082N	CGCGCGCCATGGAGATTTTCTCCTTTGATGAAAAAC	NcoI
	h	FwNMC2095N	CGCGCGCATATGGGTTACCTATCAAATAATTTACCT	NdeI
		RvNMC2095E	CGCGCGGATATCAGTTTTTTAATGACATCGGTCTAT	EcoRV
Preparation of plasmids for overexpression				
MafI$_{MGI-3}$		FwMMC2085 fw	GCGCGCCATATGATGAAAAAAAATATTTTTCAC	NdeI
		Re NMC2084	GCGCGCCTCGAGTTTTCCCAGTGGCTCAAAATTAATTGTTTC	XhoI
MafI2$_{MGI-2}$		Fw0598	GCGCGCCATATGAATATATTACCAAGCTGGCTGCGAGTCGG	NdeI
		Rev0598	GCGCGCCTCGAGCGCTTGCGAAATTAATTCCTTTCTCC	XhoI

Toxic domain of MafB2$_{MGI-2}$	Fw0597ct	GCGCGC<u>CCATGG</u>*ACTACAAAGACGATGACGACAAG*GGG ACTAAAATTCATGATGGAGCTCAAGGGAAAC	NcoI, Flag-tagged
	Rev0597ct	GCGCGC<u>CCGCGG</u>CTATTGCACCTTTTTAATGGTTTTAGGG	SacII
Toxic domain of MafB$_{MGI-3}$	Fw2084ct	GCGCGC<u>CCATGG</u>*ACTACAAAGACGATGACGACAAG*AAC AAGCCAGTTGTTAAATC	NcoI, Flag-tagged
	Rev2084ct	GCGCGC<u>CCGCGG</u>TCATTTGACAAAATATCCAGAATGA	SacII
Cloning procedures			
Amplification of the *araBAD* promoter	FwArac	GCGCGC<u>AGTACT</u>ATCGATGCATAATGTGCC	ScaI
	RevArac	GCGCGC<u>CCGCGGT</u>CCTACTCAGGAGAGCGT	SacII

[a] Letters correspond to the PCR fragments indicated in Figure 1.
[b] Primer names are based on the corresponding gene locus in strain FAM18.
[c] Restriction sites (underlined) used for cloning and sequences for protein tags (italics) for included in primers sequences are indicated.
